# The Association Between Vitamin D Deficiency and Nondiabetic Retinopathy in the American Population: National Health and Nutrition Examination Survey 2005–2008

**DOI:** 10.1155/bmri/2828949

**Published:** 2025-09-02

**Authors:** Chunyan Lei, Feipeng Jiang, Li Zhang, Qibo Ran, Yun Zhang, Meixia Zhang

**Affiliations:** ^1^ Department of Ophthalmology, West China Hospital, Sichuan University, Chengdu, China, scu.edu.cn; ^2^ Research Laboratory of Macular Disease, West China Hospital, Sichuan University, Chengdu, China, scu.edu.cn

**Keywords:** National Health and Nutrition Examination Survey, NHANES, nondiabetic retinopathy, vitamin D deficiency

## Abstract

**Objectives:** Retinopathy is a vascular endothelial injury disease that can occur in individuals without diabetes. The prevalence rates of nondiabetic retinopathy (NDR) vary from 6% to 13.6% among individuals. Vitamin D deficiency (VDD) is common worldwide, and studies indicate that the overall prevalence rate of VDD in US adults is 41.6%. Ample evidence indicates an inconsistent relationship between VDD and diabetic retinopathy, but the association between VDD and NDR remains limited.

**Design:** We conducted a population‐based, cross‐sectional study.

**Settings:** The study was conducted using data from the National Health and Nutrition Examination Survey 2005–2008.

**Participants:** A total of 4076 adults (52.71% female) with a mean age of 55.79 ± 11.72 years were included.

**Primary and Secondary Outcomes:** The primary outcome was the association between vitamin D and NDR, while there was no secondary outcome.

**Results:** Retinopathy was detected in 309 nondiabetic subjects (7.6%), while VDD was detected in 19.36% of the NDR participants. In the univariate analysis, significant associations were found between systolic blood pressure (odds ratio [OR]: 1.02; 95% confidence interval (CI): 1.00, 1.04; *p* = 0.0227), physical activity group (OR: 0.63; 95% CI: 0.51, 0.78; *p* = 0.0001), and retinopathy in the nondiabetic participants. Logistic regression analysis revealed that after adjusting for other confounders, no statistically significant association between vitamin D concentration and NDR severity was found (OR: 1.02; 95% CI: 0.97; 1.06; *p* = 0.9024). Similarly, smooth curve fitting could not find any trend between the two. Moreover, these results were consistent with the results of taking vitamin D (quartile) as a categorical variable (*p* for trend was 0.8401).

**Conclusion:** In the present study, serum vitamin D concentrations within the observed range were not significantly associated with NDR risk in the nondiabetic US population, indicating that vitamin D status is unlikely to be a primary determinant of subclinical microvascular pathology in nondiabetic adults.

## 1. Introduction

Retinopathy signs—including microaneurysms, hard exudates/cotton wool spots, and/or retinal hemorrhage—are increasingly detected in individuals without clinically diagnosed diabetes [1–5]. Prevalence studies of nondiabetic retinopathy (NDR) demonstrate significant geographic variation: Among Caucasians over 40 years, rates range from 7.8% to 12.5%, while the overall weighted prevalence in nondiabetic Americans aged 40 years and older is 6.7% [3]. Asian populations exhibit notably disparate rates, from 5.6% in the Singapore Indian Eye Study [2] to 13.6% in the Handan Eye Study [5]. Prospective population‐based studies further document that the incidence rate of retinopathy ranges from 6% over 5 years (Beaver Dam Eye Study; participants aged 55–74 years without diabetes) [6] to 7% over 9 years (Hoorn Study in the Netherlands; nondiabetic individuals aged 50–74 years) [7]. Clinically, NDR may serve as an early indicator of ocular pathologies such as retinal vein occlusions and could signal systemic vasculopathy, including hypertension [8]. Despite these implications, NDR remains frequently overlooked by both ophthalmologists and patients. Given its substantial population burden and association with underlying microvascular dysfunction, heightened clinical recognition of NDR is imperative.

Compared with diabetic retinopathy (DR), the risk factors for NDR remain less clearly characterized. Population‐based studies have established associations of NDR with impaired glucose tolerance [2, 9, 10], components of metabolic syndrome [11], hypertension [2, 9, 10, 12–14], and other cardiovascular risk factors [1, 8]. Analysis of the 2005–2008 National Health and Nutrition Examination Survey (NHANES) by Zhu et al. [3] further identified elevated blood pressure, increased glycosylated hemoglobin A1c (HbA1c), and stroke history as significant correlates of NDR. Critically, this study demonstrated that HbA1c and blood pressure values independently predicted NDR risk [3], suggesting retinal microvascular damage in nondiabetic individuals may reflect underlying glucose dysregulation and hemodynamic dysfunction. Collectively, these findings position NDR as a potential preclinical biomarker for systemic vasculopathy.

Vitamin D deficiency (VDD) is prevalent in the United States, affecting 41.6% of adults [15]. Substantial evidence implicates VDD in vascular endothelial injury pathogenesis, particularly in diabetes [16–18] and cardiovascular diseases [19–21]. Over the past two decades, research has rigorously examined vitamin D′s association with microvascular function. Microvascular dysfunction—a systemic disorder involving inflammatory pathways, endothelial impairment, and coagulation abnormalities—consistently correlates with reduced vitamin D levels across cardiac, cerebral, and renal systems [22]. Mechanistic studies demonstrate that vitamin D preserves microvascular integrity by enhancing antioxidant activity, upregulating vascular eNOS expression [23], and countering IL‐17‐mediated inflammatory cascades [24]. Clinically, VDD independently associates with increased arterial stiffness and endothelial dysfunction in conductance and resistance blood vessels, irrespective of conventional risk factors [25, 26].

This is consequential given the established correlation between endothelial dysfunction severity and cardiovascular event risk [27]. Vitamin D confers cardioprotection through the renin–angiotensin–aldosterone system (RAAS) inhibition, oxidative stress reduction, and anti‐inflammatory actions [28]—notably crucial as RAAS dysregulation precipitates metabolic syndromes including hypertension, diabetes, cardiovascular, and renal diseases [29]. Consistent observational data reveal inverse correlations between circulating vitamin D concentrations and RAAS activity [30–32], indicating vitamin D′s blood pressure–lowering effects via endothelial improvement [33]. Collectively, these findings suggest that VDD may contribute to NDR pathogenesis. We, therefore, leveraged NHANES data to investigate the VDD‐NDR relationship, addressing a critical evidence gap in microvascular pathophysiology.

## 2. Materials and Methods

### 2.1. Data Source

The NHANES is a national population‐based, cross‐sectional survey conducted every 2 years to collect and analyze data in the United States according to a complex, stratified, multistage probability cluster design. The datasets of the NHANES from 2005 to 2008 were merged in the present analysis. The NHANES protocols were approved by the National Center for Health Statistics research ethics review board. In accordance with the tenets of the Declaration of Helsinki, all participants provided written informed consent.

### 2.2. Study Population

Only surveyed participants aged 40 years and older had 45‐degree nonmydriatic digital images of the retina taken as described [34], and the images could be used for retinal grading. Therefore, the study population included only nondiabetic patients aged ≥ 40 years from NHANES 2005–2008. NDR was defined as retinopathy signs (e.g., microaneurysms, hard exudates/cotton wool spots, and/or retinal hemorrhage) in individuals without clinically diagnosed diabetes [1–5]. Individuals without complete retinopathy grading, pregnant women, individuals with diabetes mellitus, any history of eye surgery, other specific nondiabetic diseases (e.g., retinal artery occlusion, branch retinal vein occlusion, and central retinal vein occlusion), and missing vitamin D values were excluded from the analysis.

### 2.3. Assessment of Retinopathy

Two 45‐degree nonmydriatic digital retinal images per eye (one centered on the macula and one centered on the optic nerve) were obtained from participants aged 40 years and older using a Canon Nonmydriatic Retinal Camera CR6‐45NM, following the standardized NHANES retinal imaging protocol [34]. The protocol underwent rigorous quality control, including standardized technician training, systematic image review by experienced graders at the University of Wisconsin, and adjudication of discordant grades by a third grader or adjudicator to ensure consistency [35]. Fundus photographs were graded at the University of Wisconsin Ocular Epidemiologic Reading Center, Madison. The quality control of image capturing and grading has been described in detail elsewhere [36]. Retinopathy severity was determined by assessment of the retinopathy level on the basis of the NHANES grading protocol of the worse eye. Therefore, the final data analysis only included the worse eye. The Early Treatment Diabetic Retinopathy Study (ETDRS) grading standard was used to assess retinopathy severity: no retinopathy (Levels 10–13), minimal‐to‐mild nonproliferative retinopathy (Levels 14–31), moderate‐to‐severe nonproliferative retinopathy (Levels 41–51), and proliferative retinopathy (level > 60) in NHANES 2005–2008. We classified the NDR into two categories: no retinopathy and any retinopathy, and the latter included minimal‐to‐mild nonproliferative retinopathy, moderate‐to‐severe nonproliferative retinopathy, and proliferative retinopathy.

### 2.4. Serum 25‐Hydroxyvitamin D (25[OH]D) Levels

Serum 25(OH)D was measured at the National Center for Environmental Health, CDC, Atlanta, Georgia, using the DiaSorin RIA Kit (Stillwater, Minnesota, United States) from NHANES 2005–2006. However, liquid chromatography–tandem mass spectrometry (LC‐MS/MS) was performed from the NHANES 2007–2008. The CDC LC‐MS/MS method has better analytical specificity and sensitivity than immunoassay methods do; therefore, we converted RIA to LC‐MS/MS equivalents for NHANES 2005–2006 (LC − MS/MS_equivalent_ = 8.36753 + 0.97012∗RIA_original_) as recommended [37]. When the serum 25(OH)D concentration was ≤ 20 ng mL^−1^ (50 nmol L^−1^), it was defined as VDD [38–41]. To improve the statistical power, serum 25(OH)D levels were classified into a quartile categorical variable: < 10 ng/mL; ≥ 10, < 20 ng/mL; ≥ 20, < 30 ng/mL; and ≥ 30, < 100 ng/mL.

### 2.5. Other Study Variables

In the present analysis, the estimated glomerular filtration ratio (eGFR) was determined via the Chronic Kidney Disease Epidemiology Collaboration (CKD‐EPI) equation [42]. We recalibrated the serum creatinine values to the standardized creatinine measurements with the NHANES 2005–2006 recommended calibrations [34]. Chronic kidney disease (CKD) is characterized by an eGFR of < 60 mL/min/1.73 m^2^ and/or urine albumin–creatinine ratio ≥ 30 mg/g [43]. Race/ethnicity was classified as non‐Hispanic White, non‐Hispanic Black, Mexican American, and others. Education level was divided into less than 9th grade, Grades 9–12, and college or above. The factors mentioned above were obtained from demographic questionnaire data. The mean of three measurements of blood pressure was used for analysis. Hypertension was defined as systolic blood pressure ≥ 140 mmHg, diastolic blood pressure ≥ 90 mmHg, or the use of antihypertensive medication. In this study, glycohemoglobin referred specifically to HbA1c. Diabetes mellitus was defined as physician diagnosis or the use of insulin/diabetic tablets, or HbA1c ≥ 6.5%. Glycemia status was categorized as normoglycemia (HbA1c < 5.7%) and prediabetes (≥ 5.7% to ≤ 6.49%). Hyperlipidemia was defined as total serum cholesterol ≥ 240 mg/dL or the use of a lipid‐lowering agent. Smoking status and alcohol consumption were self‐reported. A C‐reactive protein (CRP) level of ≥ 1 mg/dL was considered to indicate a high CRP level. Self‐reported chronic heart diseases included congestive heart failure, coronary heart disease, angina, heart attack, and stroke. The above data were all obtained from medical conditions that were self‐reported by the participants. In accordance with the criteria of the World Health Organization (WHO), body mass index (BMI) was divided into three categories: normal, overweight, or obese (< 25, 25–29.9, and ≥ 30 kg/m^2^). Serum calcium was determined by the Beckman Synchron LX20 (Beckman Coulter, Brea, California). The spherical equivalent (SE) was calculated by summing the spherical dioptric power with half of the cylindrical dioptric power. Myopia was defined as the largest SE value not exceeding −1.0 D [44] combined with the best vision of no less than 20/25. At least one myopic eye was classified as a myopic subject. Myopia was further categorized based on SE values: low myopia (−3.00 D < SE ≤ −1.00 D), moderate myopia (−6.00 D < SE ≤ −3.00 D), and high myopia (SE ≤ −6.00 D) [45]. Physical activity levels were assessed using the Global Physical Activity Questionnaire (GPAQ), a validated instrument developed by the WHO for the surveillance of population physical activity patterns [46]. All participants were categorized into two groups: Those who achieved ≥ 600 MET minutes/week (equivalent to 150 min of moderate‐intensity or 75 min of vigorous‐intensity activity weekly) were classified as meeting physical activity recommendations (moderate or vigorous group), whereas those below this threshold (< 600 MET minutes/week) were designated as insufficiently active (light group) [47].

### 2.6. Statistical Analysis

The baseline characteristics of the study population are presented as the means ± standard deviations (SDs) for approximately normally distributed continuous variables and percentages for categorical variables.

In addition to gender, age, and race, we selected confounders based on their associations with the outcomes of interest or a change in effect estimate of more than 10% [48] or previous references. Logistic regression modeling was used to estimate the odds ratios (ORs) and 95% confidence intervals (CIs) for the association between vitamin D level and NDR, and smooth curve fitting was employed to briefly present the correlation between vitamin D concentration and NDR. A two‐sided *p* < 0.05 was considered statistically significant. Statistical analyses were performed using R software, Version 3.4.3 (http://www.R-project.org/, The R Foundation).

### 2.7. Participant and Public Involvement in the Research

Patients or the public were not involved in the design, conduct, reporting, or dissemination plans of our research.

## 3. Results

### 3.1. Selection of the Study Population

**Figure 1:**
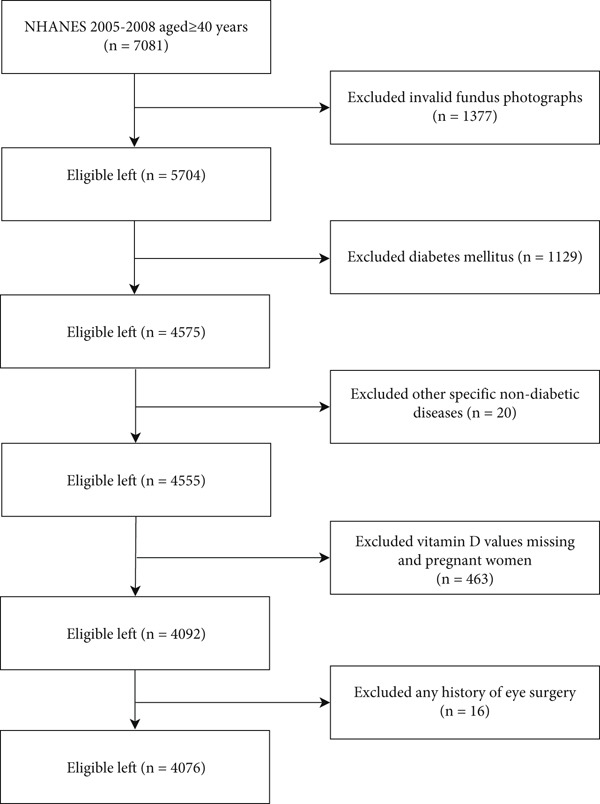
Flow chart of the selection of study population.

The analytical cohort comprised 7081 individuals aged 40 years or older who participated in the NHANES 2005–2008. Among these individuals, 3005 participants were excluded because of missing information from ungradable photographs in both eyes (*n* = 1377), diagnosis of diabetes mellitus (*n* = 1129), retinopathy due to other specific nondiabetic diseases (*n* = 20, e.g., retinal artery occlusion, branch retinal vein occlusion, and central retinal vein occlusion), missing vitamin D values and pregnancy (*n* = 463), or any history of eye surgery (*n* = 16). This left a final sample of 4076 participants for analysis (Figure 1).

### 3.2. Baseline Clinical Characteristics of the NDR

The study population included 4076 adults (52.71% female) with a mean age of 55.79 ± 11.72 years. NDR was identified in 309 subjects (7.6%), among whom VDD prevalence was 19.36%. Compared to those without retinopathy, NDR subjects were significantly older (58.43 ± 12.38 vs. 55.61 ± 11.68 years, *p* = 0.0003), had lower family income‐to‐poverty ratios (3.08 ± 1.50 vs. 3.40 ± 1.54, *p* = 0.0017), and exhibited higher systolic blood pressure (130.48 ± 21.15 vs. 124.95 ± 17.70 mmHg, *p* < 0.0001). Notably, the NDR group demonstrated higher proportions of males (57.47% vs. 46.55%), light physical activity (51.83% vs. 39.89%), hypertension (51.24% vs. 39.28%), CKD (17.79% vs. 11.78%), VDD (22.81% vs. 19.11%), and self‐reported cardiovascular disease (Table 1).

**Table 1:** Baseline characteristics of nondiabetic participants with and without retinopathy.

**Variable**	**Overall (** **N** = 4076**)**	**No retinopathy (** **N** = 3767**)**	**Any retinopathy (** **N** = 309**)**	**p** **value**
Demographics				
Age (years)	55.79 ± 11.72	55.61 ± 11.68	58.43 ± 12.38	0.0003
Male (%)	47.29%	46.55%	57.47%	0.0006
Race/ethnicity (%)				0.0512
Non‐Hispanic White	7.72%	7.58%	9.65%	
Non‐Hispanic Black	80.35%	80.79%	74.08%	
Mexican American	4.89%	4.81%	6.03%	
Others	7.04%	6.82%	10.24%	
Education level (%)				0.0006
Less than 9th grade	5.82%	5.65%	8.21%	
Grades 9–12	35.93%	35.32%	44.50%	
College or above	58.25%	59.03%	47.29%	
Income‐to‐poverty ratio	3.38 ± 1.55	3.40 ± 1.54	3.08 ± 1.50	0.0017
Smoking status (%)				0.0257
Never	48.55%	48.90%	43.63%	
Former	30.72%	30.82%	29.20%	
Current	20.73%	20.28%	27.17%	
Alcohol consumption (%)				0.0448
Never	9.95%	9.64%	14.32%	
Former	15.07%	15.07%	15.11%	
Current	74.98%	75.29%	70.57%	
Physical activity group				0.0001
Light	41.37%	39.89%	51.83%	
Moderate or vigorous	58.63%	60.11%	48.17%	
Blood pressure				
Hypertension (%)	40.08%	39.28%	51.24%	< 0.0001
Systolic (mmHg)	125.31 ± 18.00	124.95 ± 17.70	130.48 ± 21.15	< 0.0001
Diastolic (mmHg)	72.41 ± 12.53	72.32 ± 12.43	73.63 ± 13.85	0.1023
Serum lipids				
Hyperlipidemia (%)	48.50%	48.54%	47.85%	0.8392
Total cholesterol (mmol/L)	5.31 ± 1.02	5.32 ± 1.01	5.18 ± 1.08	0.0275
HDL (mmol/L)	1.41 ± 0.42	1.42 ± 0.42	1.37 ± 0.38	0.0795
LDL (mmol/L)	1.58 ± 1.08	1.58 ± 1.10	1.61 ± 0.86	0.7068
Triglycerides (mmol/L)	3.15 ± 0.89	3.15 ± 0.89	3.09 ± 0.93	0.4297
Apolipoprotein B (g/L)	1.01 ± 0.24	1.01 ± 0.24	1.00 ± 0.24	0.6115
Glucose metabolism				
Fasting glucose (mmol/L)	5.64 ± 0.62	5.63 ± 0.62	5.71 ± 0.61	0.0615
Insulin (pmol/L)	69.15 ± 67.75	67.53 ± 65.65	88.62 ± 87.18	0.0003
HbA1c (%)	5.44 ± 0.36	5.43 ± 0.36	5.50 ± 0.38	0.0016
Anthropometrics				
BMI (kg/m^2^)	28.49 ± 6.20	28.48 ± 6.24	28.57 ± 5.55	0.7985
Waist circumference (cm)	98.81 ± 14.90	98.72 ± 14.94	100.13 ± 14.24	0.1386
Renal function				
CKD (%)	12.17%	11.78%	17.79%	0.0034
eGFR	92.69 ± 21.15	92.94 ± 21.11	89.18 ± 21.40	0.0048
UACR	22.30 ± 153.55	20.62 ± 141.70	46.00 ± 270.05	0.0088
Cardiovascular history (%)				
Congestive heart failure	2.18%	2.18%	2.21%	0.9745
Coronary heart disease	3.70%	3.54%	5.90%	0.0485
Angina	2.45%	2.38%	3.55%	0.2279
Myocardial infarction	3.65%	3.61%	4.21%	0.6076
Stroke	2.95%	2.70%	6.47%	0.0005
Ophthalmic parameters				
Spherical equivalent	−0.24 ± 0.32	−0.24 ± 2.34	−0.24 ± 2.08	0.0998
Myopia severity (%)				0.1095
None	68.05%	67.05%	72.95%	
Low	19.95%	19.95%	19.05%	
Moderate	8.85%	9.05%	6.05%	
High	3.15%	3.95%	1.95%	
Serum vitamin D (ng/mL)	27.65 ± 8.65	27.73 ± 8.67	26.67 ± 8.33	0.0518
Vitamin D deficiency	19.36%	19.11%	22.81%	0.1316
Vitamin D (ng/mL) group				0.1138
< 10 ng/mL	1.01%	0.98%	1.45%	
≥ 10, < 20 ng/mL	18.35%	18.13%	21.36%	
≥ 20, < 30 ng/mL	42.25%	42.13%	43.85%	
≥ 30, < 100 ng/mL	38.39%	38.76%	33.34%	
Inflammation markers				
CRP (mg/dL)	0.41 ± 0.87	0.41 ± 0.88	0.38 ± 0.63	0.5732
Total calcium (mmol/L)	2.36 ± 0.09	2.36 ± 0.09	2.36 ± 0.09	0.5349

*Note:* All *p* values were calculated by chi‐square test.

Abbreviations: BMI, body mass index; CKD, chronic kidney disease; CRP, C‐reactive protein; eGFR, estimated glomerular filtration rate; HbA1c, glycosylated hemoglobin A1c; HDL, high‐density lipoprotein cholesterol; LDL, low‐density lipoprotein cholesterol; UACR, urine albumin–creatinine ratio.

### 3.3. Univariate Analysis for NDR

In the univariate analysis, significant associations were found between systolic blood pressure (OR: 1.02; 95% CI: 1.00, 1.04; *p* = 0.0227), physical activity (OR: 0.63; 95% CI: 0.51, 0.78; *p* = 0.0001), and retinopathy in the nondiabetic participants (Table 2).

**Table 2:** Univariate analysis for nondiabetic retinopathy.

**Characteristics**	**OR (95% CI)**	**p** **value**
Female vs. male	0.63 (0.37, 1.08)	0.0973
Age, per 1 year increase	1.03 (1.01, 1.05)	0.0755
Race/ethnicity		
Non‐Hispanic White	1.0	
Non‐Hispanic Black	0.70 (0.28, 1.73)	0.4485
Mexican American	0.97 (0.24, 3.72)	0.9718
Others	1.15 (0.35, 3.74)	0.8031
Education level		
Less than 9th grade	1.0	
Grades 9–12	0.86 (0.31, 2.35)	0.7819
College or above	0.55 (0.20, 1.48)	0.2417
Ratio of family income to poverty, per 0.1 increase	0.87 (0.74, 1.03)	0.1284
Smoking status		
Never	1.0	
Former	1.05 (0.57, 1.96)	0.8421
Current	1.51 (0.78, 2.83)	0.2112
Alcohol consumption		
Never	1.0	
Former	0.66 (0.25, 1.77)	0.4239
Current	0.62 (0.28, 1.35)	0.2384
Physical activity group		
Light	1.0	
Moderate or vigorous	0.63 (0.51, 0.78)	0.0001
Blood pressure		
Hypertension vs. absent	1.62 (0.96, 2.72)	0.0632
Systolic, per 1 mmHg increase	1.02 (1.00, 1.04)	0.0227
Diastolic, per 1 mmHg increase	1.02 (0.99, 1.04)	0.4310
BMI, per 1 kg/m^2^ increase	0.99 (0.95, 1.03)	0.9035
BMI (kg/m^2^)		
Normal (< 25)	1.0	
Overweight (≥ 25, < 30)	1.37 (0.71, 2.61)	0.3275
Obese (≥ 30)	1.16 (0.58, 2.29)	0.6582
Waist circumference, per 1 cm increase	1.00 (0.98, 1.02)	0.4791
Serum lipids		
Total cholesterol, per 1 mmol/L	0.86 (0.66, 1.12)	0.2903
Hyperlipidemia vs. normal lipids	0.96 (0.57, 1.62)	0.4330
Direct HDL cholesterol, per 1 mmol/L increase	0.75 (0.39, 1.43)	0.4023
Triglyceride, per 1 mmol/L increase	1.04 (0.75, 1.40)	0.8573
LDL cholesterol, per 1 mmol/L increase	0.92 (0.62, 1.36)	0.7052
Apolipoprotein (B), per 1 g/L increase	0.83 (0.19, 3.47)	0.8075
Fasting glucose (mmol/L)	1.26 (0.74, 2.14)	0.3715
Insulin (pmol/L)	1.01 (1.00, 1.02)	0.0998
CRP, per 1 mg/dL increase	0.94 (0.67, 1.33)	0.7880
High CRP vs. normal CRP	0.76 (0.28, 2.03)	0.6017
HbA1c, per 1% increase	1.72 (0.84, 3.50)	0.1337
HbA1c (%)		
Normoglycemia (< 5.7%)	1.0	
Prediabetes (≥ 5.7%, ≤ 6.49%)	1.53 (0.87, 2.64)	0.1352
Renal function		
CKD	1.61 (0.81, 3.20)	0.1665
eGFR	0.98 (0.97, 1.00)	0.1773
UACR	1.00 (1.00, 1.00)	0.2982
Self‐reported		
Congestive heart failure vs. absent	1.02 (0.17, 5.86)	0.9879
Coronary heart disease vs. absent	1.70 (0.55, 5.23)	0.3518
Angina/angina pectoris vs. absent	1.52 (0.36, 6.20)	0.5674
Heart attack vs. absent	1.17 (0.31, 4.27)	0.8059
Stroke vs. absent	2.48 (0.83, 7.40)	0.1027
Spherical equivalent	1.05 (0.99, 1.11)	0.1195
Myopia		
Without	1	
Low	0.83 (0.65, 1.06)	0.1528
Middle	0.61 (0.34, 1.08)	0.0927
High	0.50 (0.18, 1.38)	0.1815
Vitamin D, per 1 ng/mL increase	0.98 (0.95, 1.01)	0.3507
Absent vs. vitamin D deficiency	0.79 (0.42, 1.47)	0.4728
Vitamin D (ng/mL) Group 1		
< 30	1.0	
≥ 30	0.78 (0.45, 1.35)	0.3985
Vitamin D (ng/mL) Group 2		
< 10	1.0	
≥ 10, < 20	0.77 (0.07, 7.37)	0.8326
≥ 20, < 30	0.68 (0.07, 6.27)	0.7449
≥ 30, < 100	0.56 (0.05, 5.22)	0.6203
Total calcium, per 1 mmol/L increase	0.64 (0.03, 11.00)	0.7672

Abbreviations: BMI, body mass index; CI, confidence interval; CKD, chronic kidney disease; CRP, C‐reactive protein; eGFR, estimated glomerular filtration rate; HbA1c, glycosylated hemoglobin A1c; HDL, high‐density lipoprotein cholesterol; LDL, low‐density lipoprotein cholesterol; OR, odds ratio; UACR, urine albumin–creatinine ratio.

### 3.4. Relationships Between Vitamin D Concentration and NDR in Different Models

**Figure 2:**
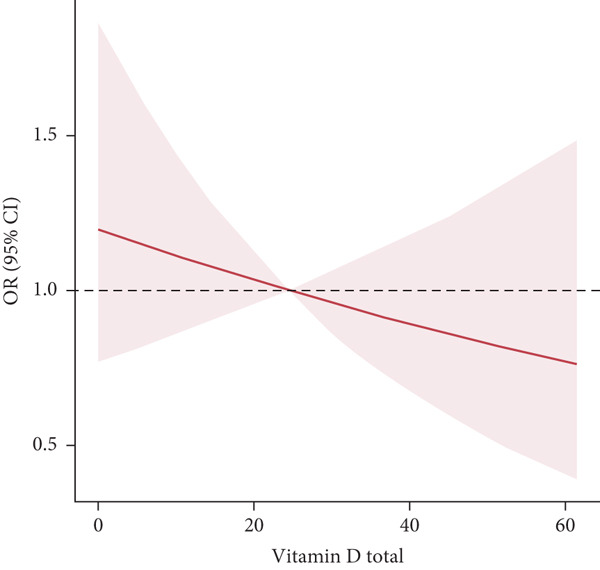
Smooth curve fitting of vitamin D and nondiabetic retinopathy. The relationship between the concentration of vitamin D and retinopathy severity (OR). The red shaded area represents the 95% confidence interval, and the solid red line represents the regression line. A linear relationship between them was detected after adjusting for sex, age, race/ethnicity, stroke, smoking status, alcohol consumption, physical activity, education level, hypertension, glycosylated hemoglobin A1c (%), hyperlipidemia, myopia, and chronic kidney disease.

Logistic regression analysis, after adjusting for sex, age, race/ethnicity, stroke, smoking status, alcohol consumption, physical activity, education level, hypertension, HbA1c, hyperlipidemia, myopia, and CKD, revealed no association between vitamin D concentration and NDR (OR: 1.02, 95% CI: 0.97, 1.06; *p* = 0.9024) (Table 3). Similarly, smooth curve fitting revealed no discernible trend between the two (Figure 2). These results were consistent with the analysis treating vitamin D concentration as a categorical variable (quartiles; *p* for trend = 0.8401), though the OR for retinopathy showed a nonsignificant decreasing trend with increasing vitamin D concentrations.

**Table 3:** Multiple adjusted logistic regression models for nondiabetic participants.

**Exposure**	**Nonadjusted**	**Adjust I**	**Adjust II**
Vitamin D (ng/mL)	1.00 (0.97, 1.03) 0.3521	0.98 (0.95, 1.01) 0.5798	1.02 (0.97, 1.06) 0.9024
Absent vs. vitamin D deficiency	0.81 (0.44, 1.50) 0.4687	0.87 (0.45, 1.66) 0.6392	0.93 (0.50, 1.75) 0.8653
Vitamin D (ng/mL) Group 1			
< 30	1.00	1.00	1.00
≥ 30	0.80 (0.47, 1.37) 0.3953	0.84 (0.47, 1.48) 0.5589	0.95 (0.55, 1.65) 0.8592
Vitamin D (ng/mL) Group2			
< 10	1.00	1.00	1.00
≥ 10, < 20	0.77 (0.09, 7.35) 0.8295	0.86 (0.10, 8.41) 0.8876	0.90 (0.15, 8.50) 0.9285
≥ 20, < 30	0.70 (0.09, 6.30) 0.7418	0.76 (0.09, 7.65) 0.8243	0.87 (0.15, 8.20) 0.8988
≥ 30, < 100	0.58 (0.07, 5.25) 0.6179	0.66 (0.08, 6.82) 0.7358	0.85 (0.12, 7.90) 0.8827
*p* *for trend*	0.3350	0.5102	0.8401

*Note:* The nonadjusted model adjusts for none. The Adjust I model adjusts for gender, age, and race/ethnicity. The Adjust II model adjusts for gender, age, race/ethnicity, stroke, smoking status, alcohol consumption, physical activity, education level, hypertension, glycosylated hemoglobin A1c, hyperlipidemia, myopia, and chronic kidney disease.

## 4. Discussion

Emerging evidence indicates that VDD is associated with an increased risk of multiple ophthalmic pathologies, including uveitis [49–51], age‐related macular degeneration (AMD) [52], DR [53–56], and ocular surface disorders [57]. While the VDD‐DR association is well documented, our study extends this paradigm to NDR—a preclinical biomarker of systemic vasculopathy. Mechanistically, vitamin D demonstrates protective effects on the retina and retinal pigment epithelium against oxidative stress and inflammation in hyperglycemic environments [58]. Given NDR’s role in capturing early microvascular changes and its substantial prevalence in contemporary populations [3], it represents a valuable model for investigating vitamin D’s vasculoprotective potential. To the best of our knowledge, this constitutes the first population‐based analysis examining the VDD‐NDR relationship using NHANES data.

Mounting epidemiological evidence suggests that insufficient or deficient vitamin D status may increase the risk of CVD [59]. Supporting this, a meta‐analysis of up to 180,000 individuals [60] and thousands of events indicated that low vitamin D status is associated with a significant increase in the risk for hypertension, cardiovascular events, and cardiovascular mortality. Given retinopathy′s established role as a microvascular indicator of adverse cardiovascular profiles, our findings warrant comparison with Zhu et al.′s NHANES 2005–2008 analysis identifying sex, blood pressure, HbA1c, and stroke history as NDR risk factors [3]. While both studies utilized identical NHANES 2005–2008 data, the present univariate analysis exclusively revealed associations between systolic blood pressure, physical activity, and NDR. This divergence is likely attributable to our exclusion criteria (omitting subjects with missing vitamin D data or ocular surgery history) and methodological distinctions in statistical approaches and analytical software.

Existing clinical evidence consistently suggests that VDD is a significant risk modifier for DR pathogenesis, which is supported by mechanistic studies demonstrating the protective effects of vitamin D supplementation against DR progression [16, 18, 54, 56, 61–65]. This association is biologically plausible given the established role of VDD in exacerbating retinal oxidative stress through dysregulation of antioxidant defense systems [66–68], coupled with diabetes‐induced dysfunctions in retinal vitamin D metabolism, particularly altered 1‐*α*‐hydroxylase activity under hyperglycemic conditions that disrupt local 1,25(OH)_2_D, the active form of vitamin D synthesis [69]. Notably, retinal vitamin D receptors mediate critical regulatory effects on the RAAS, whose pathological activation in diabetes accelerates microvascular damage [38, 70, 71]. In contrast, our analysis revealed no statistically significant association between systemic vitamin D levels and NDR severity. This dichotomy may stem from three interrelated mechanisms: (1) Pathway specificity: While DR evolves through well‐characterized microvascular pathways potentiated by diabetic metabolic derangements (enhanced RAAS signaling and chronic hyperglycemia‐induced oxidative stress), NDR pathogenesis likely involves distinct etiological cascades that are less dependent on vitamin D–mediated regulation. Retinal cells in nondiabetic contexts may exhibit differential sensitivity to systemic VDD due to alternative redox homeostasis mechanisms. (2) Tissue metabolic compartmentalization: Emerging evidence suggests that ocular vitamin D levels are poorly correlated with systemic vitamin D concentrations [72, 73], implying tissue‐specific metabolic autonomy. In diabetic retinas, localized 1,25(OH)_2_D synthesis impairments synergize with systemic VDD to amplify damage, whereas nondiabetic retinal tissues may maintain compensatory metabolic plasticity to buffer systemic vitamin D fluctuations. (3) Temporal effect modulation: VDD microvascular impacts may manifest predominantly in advanced disease stages, as evidenced by its stronger association with cardiocerebrovascular endpoints than early retinopathy [74–79]. In the initial phases of NDR, confounding factors (hemodynamic stressors, electrolyte imbalances [75, 76], or genetic polymorphisms in vitamin D receptor pathways [80]) may obscure measurable VDD effects.

Methodologically, our null findings could reflect insufficient adjustment for hypertension‐mediated confounding factors given vitamin D′s known RAAS inhibitory properties [81, 82] and blood pressure′s established correlation with NDR in univariate analysis. The predominant role of glycemic dysregulation in DR pathogenesis may further eclipse subtler VDD effects in normoglycemic populations.

The lack of standardized criteria for defining VDD in the existing literature may result in inconsistent interpretations and skewed analytical outcomes. Multiple international and national clinical guidelines define VDD as a serum 25(OH)D level ≤ 20 ng/mL (50.0 nmol/L) [39–41]. The American Institute of Medical Research reported that serum 25(OH)D levels below 30 nmol/L (12 ng/mL) indicate VDD [83, 84]. At the cellular level, a 25(OH)D level of ≤ 20 ng/mL is associated with reduced activation of the vitamin D receptor in retinal tissues [63, 74]. Therefore, we adopted a 25(OH)D level of ≤ 20 ng/mL as the VDD. Compared with the ≤ 20 ng/mL cutoff, the < 12 ng/mL threshold represents a more extreme state of deficiency. While our study focused on the general VDD status (≤ 20 ng/mL) to capture a broader range of individuals at risk of preclinical systemic vascular disease, such as NDR due to VDD, the < 12 ng/mL group would only include those with the most severe form of deficiency. However, future research could explore the specific characteristics and health outcomes of this severely deficient subgroup in more detail.

The advantages of this study include the inclusion of a large number of population‐based national samples, comprehensive risk assessment, a large proportion of gradable fundus images, the ability to diagnose binocular retinopathy, and the use of standardized photographic grading protocols. Although this was a historical cross‐sectional study, we employed strict statistical adjustment to minimize any residual confounders. There are several potential limitations worth considering. First, these findings should be interpreted with caution, and the statistical insignificance between VDD and NDR risk needs confirmation from further longitudinal studies. Second, the data of NHANES 2005–2008 were from more than 10 years ago and might not be representative of today’s society. Third, the generalizability of the results may be limited, as the study population was all American. Fourth, as with all cross‐sectional designs, our study may retain residual confounding from unmeasured variables such as ultraviolet radiation exposure (sunlight), medication use, and dietary vitamin D intake, owing to the absence of these parameters in the NHANES database, even after comprehensive adjustment for recognized confounders. Fifth, different vitamin D assessment methods were used in different waves of the NHANES, which might lead to misclassification bias in the exposure data.

Additionally, the use of 45‐degree nonmydriatic imaging, while practical for large‐scale screening, has limitations compared to 7‐field fundus imaging, which offers broader retinal coverage (including peripheral regions) and may enhance detection of peripheral lesions [85]. However, 45‐degree imaging provides critical advantages for population‐based studies, including noninvasiveness and avoidance of pharmacologic dilation [86]. Notably, our adoption of two images per eye (centered on the macula and optic nerve) aligns with evidence that dual‐field 45‐degree imaging achieves high sensitivity (95.7% for any DR) and specificity (92.3% for clinically significant retinopathy) [86]. This approach partially offsets coverage limitations by capturing key regions (optic nerve and macula) vital for retinopathy assessment [85]. Coupled with NHANES’ rigorous grading protocols, including adjudication of discordant grades, this standardized dual‐image method supports the reliability of our retinopathy classifications.

## 5. Conclusions

In conclusion, serum vitamin D levels within the observed range were not significantly associated with NDR risk in the nondiabetic US population, indicating that vitamin D status is unlikely to be a primary determinant of subclinical microvascular pathology in nondiabetic adults. More epidemiological evidence is needed to investigate the associations between micronutrient levels and NDR risk.

## Ethics Statement

This study involves human participants. The survey was approved by the National Center for Health Statistics (NCHS) ethics review board. The NCHS IRB/ERC protocol number in this survey covers 2005–2008 (Protocol #2005‐06 and Continuation of Protocol #2005‐06). The participants provided informed consent to participate in the study before taking part.

## Consent

The authors have nothing to report.

## Conflicts of Interest

The authors declare no conflicts of interest.

## Author Contributions

Conceptualization: Chunyan Lei, Meixia Zhang; data collection: Li Zhang, Qibo Ran; formal analysis: Chunyan Lei, Feipeng Jiang; validation: Yun Zhang; supervision: Meixia Zhang; writing—original draft: Chunyan Lei; writing—review and editing: Chunyan Lei, Feipeng Jiang, Li Zhang, Qibo Ran, Meixia Zhang. Chunyan Lei and Feipeng Jiang contributed equally to this work.

## Funding

This work was supported by the Sichuan Provincial Science and Technology Support Project (No. 2024YFFK0303 and 2025ZNSFSC0698) and the 1·3·5 projects for Artificial Intelligence, West China Hospital, Sichuan University (ZYAI24053).

## Data Availability

Data are available in a public, open‐access repository. The NHANES is a multistage, ongoing, complex cross‐sectional health examination and survey designed to collect the health data of the US noninstitutionalized civilian population. The data supporting reported results can be freely available from the NHANES website public archive, accessible at NHANES questionnaires, datasets, and related documentation repository (https://wwwn.cdc.gov/nchs/nhanes/Default.aspx).
